# Medical Opinion and Movement

**Published:** 1910-11-19

**Authors:** 


### Medical Opinion and Movement.

### Typhoid Convalescence.

In the course of an epidemic of typhoid fever in a regiment of artillery at Kokura (Japan) Drs. Tsuzuki and Ishida carried out a series of researches with the idea of hastening the disappearance of the typhoid bacilli from the fæces of convalescents so as to prevent the persistence of typhoid bacilli "carriers." With this object they tried the effects of potassium iodide and arsenic. Those patients who had completely lost all fever for two or three weeks were divided into three groups: one group were given potassium iodide, a second group were given arsenic in the form of Fowler's solution, and the third received no special treatment at all. The fæces and urine were examined at frequent intervals. Those receiving the potassium iodide lost all traces of the typhoid bacilli on the average in forty-two days, those having arsenic in thirty-four days, and those having no treatment in fifty-nine days. So that the treatment appeared to have a definite effect in hastening the disappearance of the bacilli, and the arsenic was more efficacious than the iodide of potassium. Moreover, those who were untreated suffered for the most part from obstinate constipation, requiring enemata every two or three days, and they did not recover their strength and appetite so rapidly.

### Pyloric Stenosis and Radioscopy.

In a contribution to the *Berliner Klinische Wochenschrift*, Dr. J. Tornai demonstrates a method by which radioscopy may be used not only to show the presence and localisation of a stenosis in the digestive tube, such as pyloric stenosis, but also to measure the degree of stenosis. For this purpose he uses glutinised capsules containing subnitrate or carbonate of bismuth in three different sizes of 6, 8, and 10 millimetres diameter. The capsules are of sufficient consistency to retain for some hours their shape and dimensions in the gastric juice In order to measure the degree of stenosis, the patient swallows a capsule of small calibre, and if that is seen by the $x$-rays to traverse the stenosis, a larger one is then given. If the 6-millimetre capsule passes through the stenosis but the 8-millimetre capsule is arrested, one may conclude that the stenosed aperture is more than 6 and less than 8 millimetres in diameter. If by these means it is found after a certain interval of time that a capsule, say of 8 millimetres diameter, which previously passed the stenosis is now no longer able to get through, the author is of opinion that there is ground for assuming the presence of a malignant tumour. The duration of time that the capsules remain in the stomach is also an indication of the condition of the pylorus. In a healthy stomach the duration depends to some extent upon the size of the capsules and the number of capsules ingested. Twenty capsules of 6 millimetres diameter will pass from the stomach in a couple of hours, while the same number of 10 millimetres capsules may remain three or four hours. If the capsules are retained in the stomach by a pyloric stenosis they are not destroyed till after six or eight hours, or even longer. The capsules may be used similiarly to determine a stenosis in any part of the intestine. If the small intestine is free they will be found in the cæcum about eight hours after ingestion.

### Alcoholic Compresses.

Some years ago Dr. Salzwedel recommended the application of alcoholic compresses as a local treatment for all manner of inflammatory and phlegmonous conditions, and since then this method of treatment has been increasing in popularity on the Continent. Successful results have been reported in the treatment of tuberculous peritonitis and appendicitis, and quite recently Dr. Cheinisse has published an account of twelve cases of typhoid fever in which he claims to have obtained excellent effects by these applications. Dr. S. Marino, of Bellosguardo, after an extensive use of alcoholic compresses for superficial inflammatory conditions, has more recently applied them for internal affections. He has employed them in twenty-five cases of appendicitis, all of which have recovered without operation, and in only one case was there a recurrence, which also readily yielded to the same treatment. The author has used these local compresses with equal success also in eleven other cases of peritonitis, including four cases of chronic tuberculous peritonitis, and in twelve cases of osseous and articular tuberculosis combined with injections of iodine or Bier's method of hyperæmic congestion. He advises the use of ethylic alcohol in 70 or 80 per cent. solution. If the strength is greater it tends to harden the skin and impede absorption. Several layers of gauze are impregnated with the alcohol and applied to the affected part. Over these is placed a layer of cottonwool slightly moistened, and then a layer of dry wool, and finally a piece of indiarubber cloth or oil-silk, the whole being fixed by a bandage. The compress should be renewed every two hours, and the treatment continued without intermission till all signs of inflammation have disappeared.

### Twin Birth of Monster and Living Child.

At the Académie des Sciences de Paris, M. Edmond Perrier recently reported a case under the care of Drs. Magnan and Périllat, in which a woman gave birth to an extraordinary anencephalic monster at the same time as a living child. The monster consisted of a globular mass resembling a large ostrich egg. At one extremity were attached two badly developed lower limbs, but no trace of upper limbs or of a head could be made out. The mass weighed nearly four pounds, while its living twin was some three ounces lighter. The authors consider that the occurrence of the monster was the result of a syphilitic heredity.

### Tuberculous Hydropigenous Nephritis.

Drs. Landouzy and León Bernard have published in *La Presse Médicale* a study of a form of kidney disease which, while presenting all the classical clinical manifestations of parenchymatous nephritis, is of tuberculous origin. Recent work has shown that the tubercle bacillus causes multiple and complex reactions in the region of the kidney which are manifested clinically in different forms. The onset of an infiltrating tuberculosis of the kidney is usually insidious, the first symptom to appear in most cases being œdema, which may occasionally amount to anasarca. The urine is abundant, contains albumin, and granular casts are present along with leucocytes and red blood cells in more or less large numbers. The tubercle bacillus may occasionally be detected, either by direct examination or as the result of inoculation. This association of hæmaturia with œdema is, in Dr. Bernard's opinion, peculiar to tuberculous nephritis. The title, "hydropigenous nephritis," would seem to fit the condition, and the most interesting cases are those in which it occurs primarily. Diagnosis is difficult, and death usually follows quickly. The renal tissue is permeable as regards all substances except sodium chloride. Drs. Bernard and Salomon have moreover succeeded in producing analogous lesions in the arterial systems of dogs and rabbits by inoculating them with the tubercle bacillus. Toxins did not produce the same effects, however. Treatment is very difficult, but tannin and calcium would seem to be doubly indicated in such cases by reason of the patient's renal lesion and tuberculosis.

### Cause of General Paralysis and Tabes.

Some revolutionary hypotheses on the ætiology of these two parasyphilitic affections are proposed by Dr. W. F. Robertson in the *Journal of Mental Science*. This investigator, who is pathologist to the Scottish Asylums, has found in sixteen consecutive cases of general paralysis an abundant growth on the nasal mucous membrane of a diphtheroid bacillus which he names the *Bacillus paralyticans*. To this organism, which is found in two forms, *longus* and *brevis*, he ascribes the symptoms of the disease, and he has discovered it also in the sheath of the second division of the fifth nerve and in the Gasserian ganglion. Within the brain of the paralytic, bacilli can be demonstrated in the majority of cases—sometimes in large numbers. Similarly he ascribes tabes to a special infected focus in the genito-urinary tract. Here there is usually a mixed infection of *Bacillus paralyticans* and *coli*. In early cases he believes that great benefit is to be obtained from the use of autogenous vaccines and anti-serum. He asserts that sufficient evidence has now accumulated to show that general paralysis is a venereal disease, not only by its relation to previous syphilis but also in respect of the source of this bacterial infection, for *Bacillus paralyticans* also inhabits the endometrium, whence it is transferred to the male during sexual congress. The special vulnerability of the nose is ascribed to a weakening of the tissues there by syphilitic sclerosis. As regards tabes, there is no proof, he thinks, that syphilis is a constant antecedent, and many of the characteristic phenomena can be produced in animals by the action of these neurotoxic bacilli alone. In the discussion on this paper both Dr. Robertson's facts and his inferences were severely criticised, and the researches of Dr. Lind, of Copenhagen, published in the same journal, traverse his bacteriological results completely. Organisms having the characteristics of *B. paralyticans* were occasionally found in paralytics and tabetics, but not more often than among normal individuals used as controls.

### Post-Prostatic Cystotomy.

A new operation for opening the bladder is described in the *Medical Record* by Dr. Eugene Fuller, and advantages are claimed for it in certain cases. This was devised as an after-thought from an experience during seminal vesiculotomy, an operation devised by this surgeon. A sacculated pouch of bladder presented behind the prostate during the operation was opened and a rubber drainage-tube left in. The result was so good that the same procedure by deliberate intention suggested itself as possibly useful in cases for which suprapubic cystotomy or ordinary perineal section are not suited. Post-prostatic cystotomy, done through the perineum, combines the advantages of both these operations. It avoids the neck of the bladder and gives even better drainage than perineal cystotomy. There is no danger of infecting the *cavum Retzii* or of getting a permanent suprapubic fistula. In tubercular cases there is a marked risk of secondary infection of the vesical neck after perineal section. Suprapubic cystotomy with thickened, contracted, and non-distensible bladders is often a troublesome procedure, whereas Dr. Fuller finds that it is perfectly easy to make a longitudinal incision into the base of the bladder when once the technique for the exposure of the seminal vesicles by his method has been acquired. It is for bad tuberculous or gonococcal or phosphatic ulcerations that this operation is especially indicated, and the results are described as excellent.

### Missed Labour: Placenta Prævia.

As showing what apparent impossibilities may actually happen in connection with pregnancy and labour, a unique case reported in the *Australasian Medical Journal* is interesting. A married woman with four children, aged twenty-eight, came under the observation of Dr. Windeyer when about a week over the usual duration of pregnancy. Fœtal movements had ceased a few days, and for a fortnight there had been no increase in size of the abdomen: no fœtal heart sounds could be heard, nor fœtal parts palpated per abdomen. Projecting into the cervix was found a smooth, hard mass about as large as a hen's egg, apparently continuous with a mass extending all round the cervix and preventing the palpation of the presenting fœtal part. A diagnosis of fibroid of the upper part of the cervix was made, and it was decided to wait a while before interfering, so as to have less difficulty in delivering the macerated fœtus. Two months later an endeavour was made to induce labour by bougies, but it was impossible to insert one past the tumour. A week later laparotomy was performed, and the uterus opened. It then appeared that there was a complete central placenta prævia, and that a piece of the placenta jutted out into the cervix: it was this which had been taken for a fibroid. Missed labour itself is not very common, but in the case of central placenta prævia it is incomprehensible. The only hæmorrhages during gestation had been slight losses following vaginal examination.

### A New Treatment for Hydrocele.

Autoserotherapy, or the treatment of effusions of serous fluid by injections of the fluid itself into other parts of the body, would seem to have a recognised position on the Continent among therapeutic measures. Its employment has hitherto been confined to cases of pleural and abdominal collections of serous fluid. Berthelon, of Tunis, however, reports in the *Journal de Médecine et de Chirurgie pratique* two cases in which the method has been used with benefit for the treatment of hydrocele. The first patient, a man of middle age, gouty and arterio-sclerotic, had suffered for three years from a large hydrocele. Two cubic centimetres of the fluid were removed from the tumour by a syringe and injected into the thigh, after the usual aseptic precautions had been taken. No reaction followed, though the patient continued to go about as usual. The following day there was a marked diminution in the hardness of the hydrocele, which had decreased in size by 15 millimetres. Two days later the decrease was very marked. Two cubic centimetres more of the fluid were removed and injected into the muscles of the thigh. Three days later the scrotum on the side of the hydrocele had regained its normal size. There has been no relapse since then, though the operation took place over a month before the time of writing. In a second case, a younger patient than the former, the author collected 12 c.c. of the fluid from the hydrocele, of which he injected 2 c.c. into the thigh. The hydrocele rapidly disappeared, and by the fourth day the scrotum on the affected side was of normal volume.

### Menstrual Hæmoptysis.

Theodore J. Eastman, in the *Boston Medical and Surgical Journal*, describes a number of cases in which there appeared to be a distinct connection between the menstrual periods and the onset of attacks of hæmoptysis. In some women a congestion and increased secretion of the respiratory mucous membranes is noticed about the menstrual period. This may induce various mild symptoms, such as slight stuffiness, voice changes, and slight cough; but bronchitis, marked coryza, and severe asthmatic attacks may be present. In one case a woman aged thirty-two always had severe asthmatic attacks prior to menstruation, which disappeared a few hours after the flow had started. It needs but an exaggeration of these asthmatic symptoms to end in a broken blood-vessel and hæmoptysis. This occurred in a patient, aged forty-two, who for thirteen years had attacks of hæmoptysis every four weeks. No clinical manifestations could be found to account for these bleedings, and in similar cases described by Ratjen, Buenting, and Cornet respectively the lungs and pharynx were clinically sound. In another of the author's cases a hæmorrhage occurred while playing tennis at a time when the menses were due. No flow from the womb took place at this period, though the following two were regular. Questionable slight dulness, with slight increase in breath sounds at one apex, were noticed at the time, but since then no further signs have appeared; there has been no rise of temperature, and the patient has gained in weight. The author quotes a number of cases in which hæmoptysis at the menstrual periods was accompanied by evidence of pulmonary lesions.

### Normal Changes in Endometrium.

Norris and Keene, according to *Surgery, Gynæcology, and Obstetrics*, have examined the material removed by curetting from a hundred cases operated on during the last ten years. A careful study of each patient's history was made and only such cases included in the examination as menstruated regularly and were free from fibromyoma, cysts, inflammatory lesions, etc. The authors maintain that the endometrium of the normal menstruating woman undergoes a complete and definite 28-day cycle. They divide the cycle into four periods which glide imperceptibly into each other. The first, or premenstrual period, is characterised by the endometrium taking on all the changes common to the decidua vera at an early stage, though to a less degree. The congestion is relieved as soon as free hæmorrhage occurs. This constitutes the second period. During the post-menstrual, or third, and the interval, or fourth, period, repair takes place. There are definite histological characteristics to be found in each of the periods of the cycle, and these are most marked in the pre- and post-menstrual. Slight variations are met with, however, in different individuals. The times which the various periods of the cycle last are as follows: Pre-menstrual, ten days; menstrual, four to six days; post-menstrual, three to five; and interval, ten to twelve days. The authors insist on the importance of letting the pathologist who examines the scrapings know when the last menstrual period took place. This knowledge, coupled with a satisfactory appreciation of the changes which occur in normal endometrium during the various cyclic stages, will enable him to differentiate between normal conditions and true endometritis. The authors believe that this latter disease is far less common than is usually thought to be the case. They also would classify endometritis either as acute or chronic and avoid the faulty and cumbersome nomenclature which is so often applied to the condition.

